# Development and Electrochemical Performance of a PANI-PA-PVA Hydrogel-Based Flexible pH Fiber Sensor for Real-Time Sweat Monitoring

**DOI:** 10.3390/gels11110853

**Published:** 2025-10-25

**Authors:** Shiqi Li, Chao Sun, Meihui Gao, Haiyan Ma, Longbin Xu, Xinyu Li

**Affiliations:** 1 Department of Materials and Chemical Engineering, College of Engineering, Yanbian University, Yanji 133002, China; 2023050085@ybu.edu.cn; 2 Laboratory of Catalysis for Energy and Resources, College of Sciences, Yanbian University, Yanji 133002, China; 2022010073@ybu.edu.cn (M.G.); 2023010063@ybu.edu.cn (H.M.); 3 Department of Physics, Jilin University, Changchun 130012, China; sunc344@jlu.edu.cn; 4 Department of Chemistry, College of Science, Yanbian University, Yanji 133002, China

**Keywords:** PANI-PA-PVA hydrogel, pH sensor, stretchable electrodes, sweat pH detection, wearable health monitoring

## Abstract

Real-time sweat pH monitoring offers a non-invasive window into metabolic status, disease progression, and wound healing. However, current wearable pH sensors struggle to balance high electrochemical sensitivity with mechanical compliance. Here we report a stretchable fiber-integrated pH electrode based on a polyaniline-phytic acid-polyvinyl alcohol (PANI-PA-PVA) hydrogel, which combines mechanical elasticity with enhanced electrochemical performance for continuous sweat sensing. Freeze–thaw crosslinking of the hydrogel forms a porous interpenetrating network, facilitating rapid proton transport and stable coupling with dry-spun elastic gold fibers. This wearable device exhibits an ultra-Nernstian sensitivity of 68.8 mV pH^−1^, ultra-fast equilibrium (<10 s within the sweat-relevant acidic window), long-term drift of 0.0925 mV h^−1^, and high mechanical tolerance (gel stretch recovery up to 165%). The sensor maintains consistent pH responses under bending and tensile strains, yielding sweat pH measurements at the skin surface during running that closely match commercial pH meters (sweat pH range measured in test subjects: 4.2–5.0). We further demonstrate real-time wireless readouts by integrating elastic gold and Ag/AgCl fibers into a three-electrode textile structure. This PANI-PA-PVA hydrogel strategy provides a scalable material platform for robust, high-performance wearable ion sensing and skin diagnostics.

## 1. Introduction

Sweat is an easily accessible biological fluid rich in biomarkers that reflect human physiological and pathological states at the molecular level [1,2]. Among these biomarkers, pH serves as a key indicator of metabolic equilibrium and hydrogen ion homeostasis [3]. Abnormal sweat pH correlates with diseases such as diabetes, renal disorders, and cystic fibrosis [4,5,6,7]. Deviations from normal pH disrupt oxygen transport, inhibit enzyme activity, and induce DNA damage, ultimately impairing vital organs including the kidneys, liver, and sweat glands [8,9,10,11]. The skin surface typically maintains a weakly acidic environment (pH 4.5–6.5), whereas damaged or infected tissues exhibit alkalinity (pH 7.2–8.9) due to microbial colonisation and protease activity [12]. These pH shifts correspond to distinct wound healing phases, thereby offering clinically relevant insights [13,14,15]. Consequently, continuous monitoring of sweat pH provides a non-invasive method for real-time assessment of metabolic status, disease progression, and wound recovery. Recent advances in wearable biosensing technology have enabled in situ pH monitoring of the skin, thereby supporting broader objectives such as early disease diagnosis, dynamic treatment adjustments, and personalized precision medicine. Consequently, developing reliable, flexible, and highly sensitive pH sensors is crucial for advancing non-invasive healthcare technologies.

Wearable flexible sensors have garnered significant attention due to their lightweight design, deformability, and compatibility with the dynamic human body, enabling real-time analysis of biofluids such as sweat [1,2,16]. The performance of these devices is highly dependent on the selection of sensing materials, which must exhibit high sensitivity, mechanical strength, and biocompatibility [17,18]. Given that human skin undergoes significant deformation during daily activities—ranging from approximately 30% strain on the forearm to nearly 80% strain at the elbow—developing materials capable of withstanding repeated mechanical stress without performance degradation remains paramount. Current wearable pH sensors typically rely on three material categories: inorganic semiconductors, ion-selective electrodes (ISE), and conductive polymers [19]. Inorganic semiconductors such as ITO and ZnO exhibit high carrier mobility and excellent chemical stability [20], but their inherent rigidity and high-temperature processing requirements hinder integration with soft, flexible substrates. ISEs possess high ion selectivity [21]; however, their rigid architecture and bulk structure limit miniaturisation and skin conformability. Conductive polymers, particularly polyaniline (PANI), emerge as promising candidates owing to their low cost, biocompatibility, and robust pH responsiveness within physiologically relevant ranges (pH 2–10), including typical sweat conditions (pH 4.5–7.5, dropping to approximately 3.5 during vigorous exercise) [22,23,24]. However, pristine polyaniline (PANI) exhibits poor mechanical elasticity with a fracture strain of merely 2–5%, leading to interfacial stresses, cracking, and electrochemical instability when coupled with stretchable substrates [6]. This mismatch between electrochemical functionality and mechanical compliance remains a major obstacle to achieving long-term wearable pH monitoring. Consequently, there is an urgent need to develop strategies that enhance interfacial coupling while simultaneously optimising electrochemical and mechanical properties.

To overcome these limitations, we designed a crosslinked PANI-PA-PVA hybrid hydrogel that integrates conductivity with stretchability. Phytic acid (PA), a naturally sourced hexapolyphosphate, serves both as a dopant and crosslinking agent, providing abundant hydrogen-bonding sites for constructing a robust three-dimensional polymer network [25,26]. The incorporation of polyvinyl alcohol (PVA) further enhances elasticity by forming a flexible matrix that mechanically stabilises the conductive PANI domains. The resulting hydrogel is stabilised through ionic interactions and freeze–thaw cross-linking, exhibiting a highly interconnected porous structure that effectively balances mechanical flexibility with electrochemical performance.

**Figure 1:**
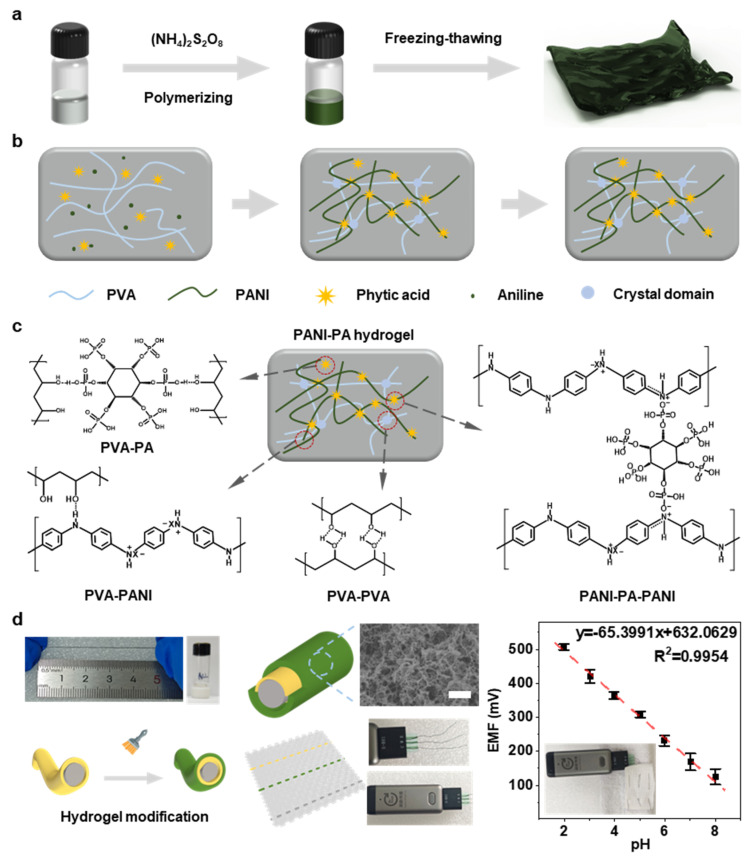
(**a**,**b**) Synthetic route of the PANI-PA-PVA hydrogel. (**c**) Schematic illustration of the cross-linked network structure, where hydroxyl groups in PA interact with hydroxyl groups in PVA and amine groups in PANI through hydrogen bonding. (**d**) Schematic and SEM image of the PANI-PA-PVA hydrogel integrated on a flexible electrode (The non-English portion in the image refers to the intelligent sensor manufacturing company).

In this study, we report a novel stretchable pH-responsive hydrogel fabricated by crosslinking aniline with phytic acid (PA) and polyvinyl alcohol (PVA) to enhance its flexibility and conductivity. As shown in Figure 1a, PA forms hydrogen bonds with both PANI and PVA, and subsequent freeze–thaw treatment yields a dense crosslinked PANI-PA-PVA hydrogel network. The material exhibits a uniform porous morphology and robust mechanical integrity (Figure 1b–d). The working electrode was fabricated by coating the hydrogel onto dry-spun elastic gold fibers, whilst the reference and counter electrodes were prepared using gold fibers coated with Ag/AgCl and bare gold fibers, respectively, forming a fiber-based three-electrode configuration (Figure 1d). The resulting fiber sensor demonstrated excellent electrochemical stability and stretchability, achieving a sensitivity of 65.6 mV·pH^−1^ at 100% elongation with minimal signal loss. When integrated into textile substrates, the device maintained stable pH detection performance in artificial sweat, with sensitivity variation of only 2.5% under 30% strain. These results highlight the potential of the PANI-PA-PVA hydrogel system as a smart, wearable, real-time pH monitoring platform for next-generation flexible electronics.

## 2. Results and Discussion

### 2.1. Characterization of PANI-PA-PVA Hydrogel

**Figure 2:**
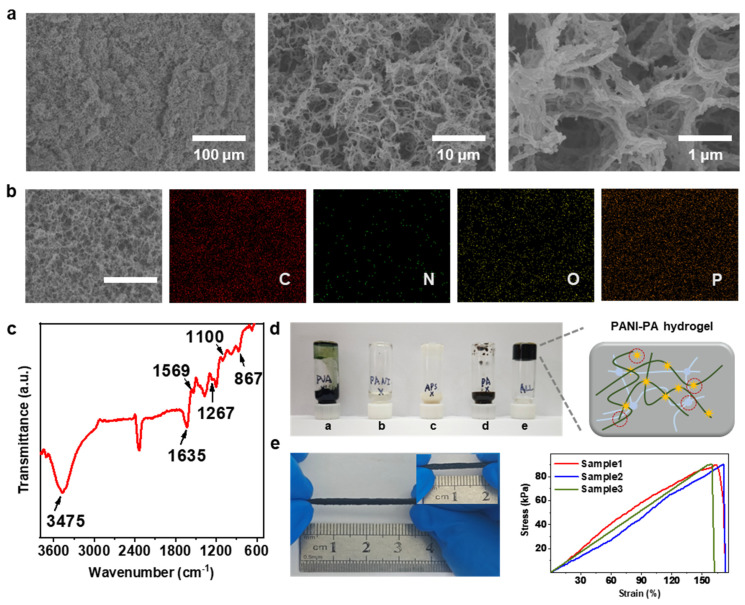
Characterization of the PANI-PA-PVA hydrogel. (**a**) SEM images at magnifications of 100 µm, 10 µm, and 1 µm, showing a uniform porous structure with interconnected pore walls. (**b**) EDS elemental mapping of C, N, O, and P, confirming the homogeneous distribution of PANI, PVA, and PA components within the hydrogel (at a 100 µm scale). (**c**) FTIR spectra of PANI and PANI-PA-PVA hydrogels, highlighting characteristic absorption peaks and hydrogen-bonding interactions. (**d**) Photographs of reactions using different reagent combinations. Vial e contained all four reactants to yield PANI-PA-PVA hydrogel. Vial a lacked PVA, vial b lacked PANI, vial c lacked APS, and vial d lacked PA (The meanings of the lines and symbols in the partial schematic diagram are identical to those in the diagram above). (**e**) Photographs of hydrogel stretching and the corresponding stress–strain curve, demonstrating its excellent elasticity and mechanical robustness.

Scanning electron microscopy (SEM) revealed that the PANI-PA-PVA hydrogel possesses a highly uniform and interconnected porous network with pore diameters predominantly in the range of 5–20 µm (Figure 2a). This hierarchical structure originates from the freeze–thaw process, in which ice crystals act as transient templates that melt upon thawing to form continuous channels. The resultant porosity is stabilized by extensive hydrogen-bonding interactions among PANI, PVA, and PA, which restrict random chain motion and maintain structural integrity under mechanical stress [27]. High-magnification SEM images (10 µm and 1 µm) reveal pore walls composed of nanoscale fibers and compact nanoparticles (200–500 nm), consistent with the morphology of previously reported PANI-based hydrogels [28,29]. The absence of rod-like PANI nanostructures further indicates that PANI chains are uniformly anchored onto the PVA backbone (see Figure S1), forming a stable interpenetrating network via hydrogen bonding. This hybrid architecture provides dual advantages: (i) mechanical robustness imparted by the fibrous PVA matrix and (ii) efficient ion-transport pathways facilitated by the porous, cross-linked framework. Together, these characteristics confer structural stability and favorable electrochemical performance, establishing the hydrogel as a promising platform for flexible and stretchable sensing.

Energy-dispersive X-ray spectroscopy (EDS) mapping confirmed homogeneous distribution of the key elements—C, N, O, and P—across the hydrogel surface (Figure 2b). The uniform dispersion of these elements demonstrates successful incorporation of PANI (C, N), PVA (C, O), and PA (P, O) into a well-blended hybrid matrix, consistent with the quantitative elemental analysis summarized in Table S1. Fourier-transform infrared (FTIR) spectroscopy further verified the chemical interactions within the composite (Figure 2c). The broad absorption band centered around 3475 cm^−1^ corresponds to N-H stretching in PANI, overlapping with the O-H stretching of PVA-evidence of extensive hydrogen bonding between the two polymers. Characteristic peaks at 1635 cm^−1^ and 1569 cm^−1^ are attributed to C=C stretching in the quinoid and benzenoid rings, confirming the expected oxidation state of PANI. The band at 1267 cm^−1^ arises from C-N stretching of aromatic secondary amines, while the out-of-plane C-H bending at 867 cm^−1^ confirms preservation of the PANI backbone. Collectively, these spectral features verify that PANI maintains its structural integrity while forming strong intermolecular interactions with PVA. The complementary EDS and FTIR analyses thus confirm the formation of a chemically stable, homogeneously integrated hydrogel network with robust interfacial coupling, providing the necessary framework for durable electrochemical sensing.

The PANI-PA-PVA hydrogel was synthesized through oxidative polymerization followed by freeze–thaw cycling, resulting in a flexible, mechanically resilient three-dimensional network. Gelation occurred only in the presence of all four essential components—APS (initiator), PVA (polymeric backbone), aniline (conductive monomer), and PA (dopant/cross-linker)—as demonstrated by the inverted-vial test (Figure 2d). Omission of any single component prevented gel formation, underscoring the indispensable role of each constituent in forming a cohesive network. The gelation mechanism involves crystallization of PVA chains during freezing, which generates microcrystalline domains stabilized by hydrogen bonding and van der Waals interactions. These domains serve as physical cross-links, while secondary hydrogen bonding between the hydroxyl groups of PVA and the amine groups of PANI provides additional reinforcement. The resulting hydrogel displays remarkable elasticity, sustaining tensile strains up to 165% and fully recovering its original length upon release (Figure 2e). This reversible deformation behavior, arising from dynamic hydrogen bonding and entropic elastic recovery, underpins the mechanical durability required for long-term wearable applications. Accordingly, the optimized PANI-PA-PVA hydrogel offers an excellent balance of stretchability, stability, and electrochemical responsiveness, making it a robust candidate for next-generation pH sensing in dynamic, real-world environments.

### 2.2. Sensing Performance of PANI-PA-PVA Hydrogel

The electrochemical performance of the PANI-PA-PVA hydrogel electrodes was assessed in terms of potential response, selectivity, and stability. Prior to measurements, the electrodes were activated by equilibration in PBS buffer (pH = 7.0), ensuring stable baseline conditions. Potentiometric testing was then carried out across standard PBS buffers spanning pH 2.0–9.0, a range that fully encompasses the physiological pH window of human sweat (typically pH 4.0–7.0).

**Figure 3:**
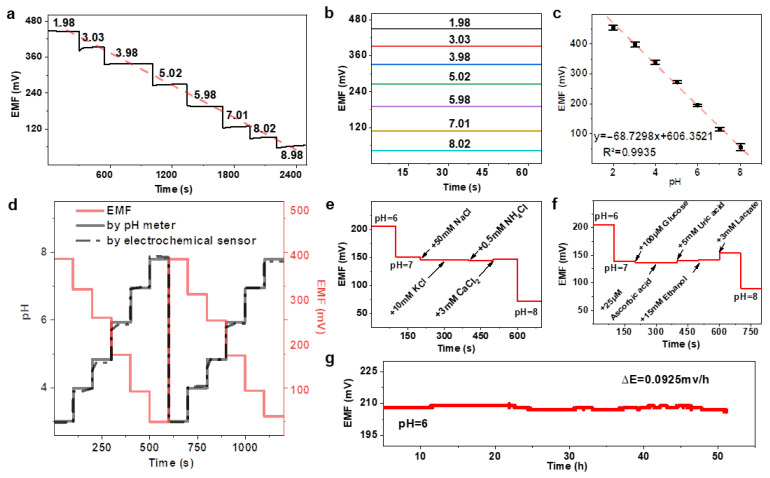
Electrochemical sensing performance of the PANI-PA-PVA hydrogel. (**a**) Response time measurements at different pH values, showing rapid potential stabilization within ≤10 s in the acidic range. (**b**,**c**) Potential response curves and corresponding calibration plots, demonstrating a linear super-Nernstian sensitivity of ~68.7 mV pH^−1^. (**d**) Comparison of hydrogel-based measurements with a commercial pH meter, confirming excellent consistency across physiological pH ranges. (**e**,**f**) Ion and biomolecule interference tests, showing minimal disturbance from Na^+^, K^+^, and NH_4_^+^, with slight deviations under Ca^2+^ and lactate. (**g**) Long-term stability evaluation at pH 6, indicating minimal potential drift (0.0925 mV h^−1^) over continuous operation. EMF (electromotive force).

The PANI-PA-PVA electrode exhibited a pronounced potentiometric response across the tested pH range, following five tests, the PANI-PA-PVA hydrogel electrode exhibited an average linear slope of 68.7 mV pH^−1^ and an excellent correlation coefficient (R^2^ = 0.9935) (Figure 3a–c). As shown in Table S2, the PANI-PA-PVA hydrogel exhibits higher sensitivity and faster response. In contrast, bare gold electrodes showed negligible sensitivity and no clear linearity (Figure S2), underscoring the crucial role of the hydrogel coating. Notably, the observed slope exceeds the theoretical Nernstian value of 59 mV pH^−1^ for polyaniline at 25 °C, demonstrating a super-Nernstian response (Figure S3). This enhanced sensitivity originates from the hydrogel’s hierarchical architecture. The three-dimensional porous framework (5–20 µm pores) generated during freeze–thaw cycling provides abundant and efficient proton transport channels, minimizing diffusion barriers. Simultaneously, quinone/benzene redox isomerization in PANI, reinforced by hydrogen bonding with PVA hydroxyl groups, promotes fast proton adsorption and electron transfer at the hydrogel-electrode interface. The synergy between structural design and molecular interactions accounts for the superior proton sensing performance. To verify accuracy, the electrode response was benchmarked against a conventional pH meter (Figure 3d). The hydrogel sensor tracked stepwise pH variations (pH 3-8-3) with high fidelity, maintaining consistency across the physiologically relevant range of human sweat. These findings confirm that the PANI-PA-PVA hydrogel enables reliable, real-time monitoring with accuracy comparable to standard instrumentation, validating its potential for in situ wearable applications.

The electrode also exhibited exceptionally rapid response dynamics. Across three measurements, the average response time was ≤120 s at pH 6, ≤100 s at pH 7, and ≤150 s at pH 8 (Figure S4). Notably, in the acidic sweat-relevant range (pH 4–6), equilibration occurred in under 120 s. Such response times are significantly faster than those of conventional solid-state electrodes. Moreover, compared to PANI-only coated electrodes at the same pH, the PANI-PA-PVA hydrogel electrode exhibits a faster response rate (Figure S5). This superior performance arises from the hydrogel’s hydrophilic, porous architecture, which facilitates rapid proton adsorption and transport, thereby minimizing interfacial mass transfer resistance. For wearable applications, this ultrafast response is particularly advantageous, as it enables real-time tracking of dynamic sweat pH fluctuations during physical activity, reducing signal lag and ensuring accurate assessment of the body’s physiological state.

To evaluate potential interferences from sweat constituents, the hydrogel electrode was tested against typical ions and metabolites at physiologically relevant concentrations [2] (Figure 3e,f). In PBS at pH = 7, the electrode exhibited negligible response to abundant cations including Na^+^, K^+^, and NH_4_^+^, confirming strong selectivity and ionic tolerance. In contrast, Ca^2+^ induced a small potential deviation (ΔE = 2.6 mV), attributed to its chelation with HPO_4_^2−^ in solution to form CaHPO_4_, which modifies local proton concentration. Lactate, a common sweat metabolite, produced a more pronounced fluctuation (~13 mV), consistent with its acidifying effect on the electrolyte and subsequent enhancement of electrochemical signal response. Environmental stability was also investigated under light and gas exposure (Figure S6). The electrode maintained stable performance under illumination, with only minor potential drift (ΔE = 2.6 mV). Exposure to N_2_ and O_2_ induced negligible changes (ΔE ≈ 1 mV), while CO_2_ caused a marked potential shift due to dissolution and carbonic acid formation, leading to localized pH reduction. These findings demonstrate that the electrode is highly resilient to typical environmental fluctuations, with only predictable responses under specific chemical perturbations. Long-term operational stability was assessed by continuously recording potential in PBS at pH = 6 (Figure 3g). The sensor displayed a remarkably low drift rate of 0.0925 mV h^−1^ over extended monitoring, confirming its robustness for sustained use.

In summary, the PANI-PA-PVA hydrogel electrode combines super-Nernstian response (68.7 mV pH^−1^), ultrafast equilibration (<10 s in the sweat-relevant acidic range), strong selectivity against common ions, and excellent environmental and temporal stability. This synergistic performance profile ensures reliable, real-time tracking of sweat pH, validating the hydrogel’s potential as a high-performance sensing platform for next-generation wearable electrochemical devices.

### 2.3. Mechanism and Electrochemical Characterization of PANI-PA-PVA Hydrogel

**Figure 4:**
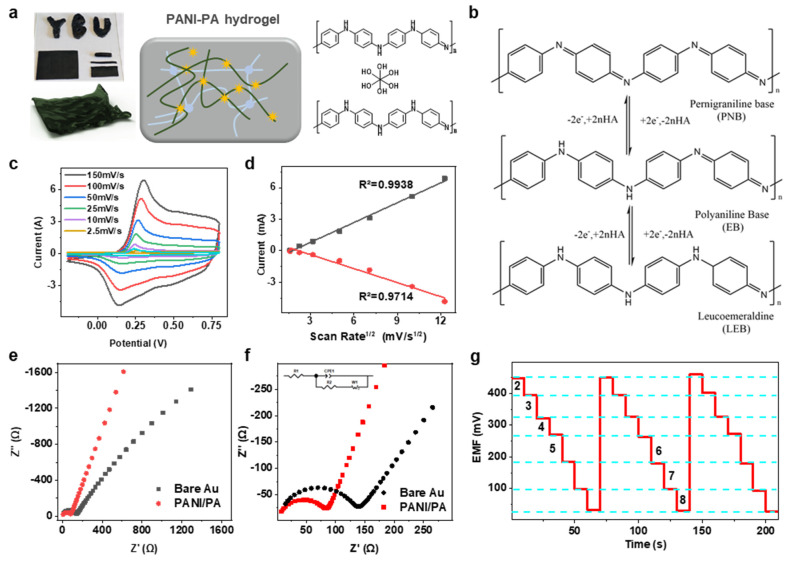
Electrochemical mechanism and performance of the PANI-PA-PVA hydrogel. (**a**) Photograph and schematic molecular structure of the hydrogel, illustrating its cross-linked porous network. (**b**) Proposed protonation-deprotonation pathways of polyaniline, underlying the pH-responsive behavior. (**c**,**d**) Cyclic voltammetry curves and corresponding diffusion characteristics in 1 M H_2_SO_4_, showing enhanced redox activity and efficient charge transfer. (**e**) Cyclic reproducibility tests across pH 2–8, demonstrating excellent stability and reversibility of electrochemical responses. (**f**,**g**) Nyquist plots comparing bare gold electrodes with PANI-PA-PVA-modified electrodes, revealing reduced charge transfer resistance and improved ion diffusion in the hydrogel system.

The PANI-PA-PVA hydrogel retained high extensibility even when molded into various three-dimensional shapes (Figure 4a), underscoring its adaptability for flexible device architectures. Electrochemical properties were further evaluated using cyclic voltammetry (CV) in 1 M H_2_SO_4_. Consistent with potentiometric data, the hydrogel sensor exhibited a high pH sensitivity of 68.7 mV pH^−1^ and ultrafast response, with mechanical robustness maintained under deformation. The super-Nernstian behavior is attributed to the acid-base doping characteristics of polyaniline, where hydrogen ions and electron pairs strongly influence protonation-deprotonation equilibria during polymerization (Figure S7). During electrochemical cycling, PANI undergoes interconversion among its characteristic redox states: leucoemeraldine salt (LS), emeraldine salt (ES), and pernigraniline (PN) (Figure 4b). The CV profile displayed an oxidation peak at 0.18 V, corresponding to the LS-ES transition (Figure 4c), while a second oxidation peak at 0.46 V was assigned to cross-linking within the PANI framework, likely induced by nitrate intermediates or peroxidation products [30]. Intermediate reduction peaks at 0.44 V and 0.50 V further suggest enhanced cross-linking or peroxidative modifications of the PANI-PA-PVA matrix [17,28]. Importantly, the hydrogel electrode exhibited significantly higher peak current density compared to pure PANI or bare Au electrodes, highlighting the role of the cross-linked, porous network in promoting charge transfer. The freeze–thaw-induced porosity increased the effective surface area, enabling more efficient ion transport and faster electrochemical kinetics. As shown in Figure 4d, peak current density scaled linearly with the scan rate from 2.5 to 150 mV s^−1^, confirming diffusion-controlled charge transfer through the porous hydrogel. At higher scan rates, anodic peaks shifted positively and cathodic peaks negatively, characteristic of electrode polarization. Reproducibility testing across three consecutive measurements within the pH 2.0–8.0 range demonstrated excellent potential reproducibility, further confirming the robust and reversible redox properties of the hydrogel [31].

To further investigate the charge transfer characteristics of PANI-PA-PVA, electrochemical impedance spectroscopy (EIS) was conducted. Polymer oxidation and reduction reactions, associated with resistive processes, were evaluated using EIS. Figure 4e shows the Nyquist plot for the PANI-PA-PVA electrode, revealing a 45° slope in the low-frequency region. This indicates capacitive processes governed by Warburg diffusion, corresponding to the oxidized state of the anthraquinone aldehyde under synthesis conditions. The charge transfer resistance (Rct) was determined by fitting the Nyquist plot (Figure 4f) to an appropriate equivalent circuit model (ECM), as shown in the inset. In this model, Z’ represents the imaginary component of the complex impedance Z*(ω), plotted against the real component Z’ to form the Nyquist plot. The equivalent circuit follows the widely used model by Amirudin and Thienrry for polymer-coated metal systems [32]. In the ECM, Rs represents the electrolyte resistance, while Rct denotes the charge transfer resistance. The constant phase element (CPE) accounts for charge accumulation at the electrolyte/polymer interface [33], influenced by the reduced electrolyte conductivity, which is typically interpreted as double-layer capacitance (CDL). W represents Warburg diffusion impedance [34]. Since Rs remains constant, it is not significant for the analysis. In PANI-based pH sensors, the polymer’s redox reaction intensifies with decreasing pH, leading to a reduction in system impedance, primarily monitored through changes in Rct.

By comparing electrochemical impedance spectroscopy (EIS) results, the charge transfer capabilities of two electrodes—bare gold and PANI-PA-PVA—were further investigated. The charge transfer resistance (Rct) for each electrode was calculated based on the diameter of the semicircle in the high-frequency region of the Nyquist plot, with values obtained through fitting using Z-view software. Upon modification, the semicircle diameters decreased, indicating improved charge transfer. The Rct values for the bare gold electrode and PANI-PA-PVA electrode were 150 Ω and 90 Ω, respectively. This reduction in Rct demonstrates that the PANI-PA-PVA-modified electrode exhibits superior charge transfer capability, which can be attributed to the high porosity and large specific surface area of the hydrogel. These properties enhance the surface utilization of PANI, create additional ion diffusion pathways, and facilitate efficient charge transfer, contributing to the excellent detection performance of the PANI-PA-PVA sensor.

Additionally, to compare the conductive properties of different formulations, the non-proton diffusion coefficient was calculated using the Randles-Sevcik equation via cyclic voltammetry (at 25 °C) [35]:

$$D={\left(\frac{S}{268,600\cdot A\cdot C\cdot n^{\frac{3}{2}}}\right)}^{2}$$

Proton diffusion coefficient formula: S: slope, n: number of electrons transferred, A: electrode area, C: proton concentration. Calculations based on cyclic voltammetry curves indicate that the proton diffusion coefficient for the PANI-PA-PVA electrode was approximately 1.13 × 10^−7^ cm^2^ s^−1^, the diffusion coefficient of the PANI-only electrode is only 6.74 × 10^−9^ cm^2^ s^−1^, while that of the PANI-PA binary hydrogel system is 1.62 × 10^−8^ cm^2^ s^−1^ (Figure S8).

In summary, the experimental data confirms that the PANI-PA-PVA hydrogel sensor efficiently monitors dynamic changes in sweat pH. This real-time pH sensing capability provides a solid technological foundation for the development of wearable sensor devices, opening up significant potential for applications in human health monitoring.

### 2.4. Performance of PANI-PA-PVA Hydrogel-Based Wearable Sensor

We first synthesised ultra-thin gold nanowires (AuNWs) with a high aspect ratio, encapsulated by oleylamine (OA), characterised by TEM (Figure S9). The elastic gold fiber was prepared via a dry spinning process, wherein AuNWs were mixed with styrene-ethylene-block copolymer (SEBS) in tetrahydrofuran (THF), followed by dry spinning under ambient conditions. The AuNWs/SEBS fiber underwent pre-stretching before immersion in a Au chemical growth solution, followed by sodium borohydride (NaBH_4_) treatment. SEM images reveal the final elastic gold electrode fiber featuring a wrinkled gold film surface (Figure S10).

To verify the consistency of elastic gold electrodes with commercially available rigid electrode materials, cyclic voltammetry was conducted using a three-electrode system with 1 M H_2_SO_4_ as the electrolyte. The cyclic voltammograms clearly exhibit the characteristic electrochemical behavior of the elastic gold electrodes in sulfuric acid, with features consistent with the response patterns of commercial rigid gold electrodes (Figure S11). Specifically, during the cathodic scan (negative current peak), a sharp and intense reduction peak appears near 0.83 V, with a peak current of approximately −4 mA. This peak corresponds to the reduction of gold surface oxides (AuO to Au). The sharp morphology of this peak indicates that the reduction process is rapid and complete, confirming the excellent electronic conductivity of the electrode material. During the anodic scan (forward current peak), a broad oxidation peak emerges around 1.2 V, corresponding to the oxidation of gold (Au to AuO), indicating the formation of an oxide layer. This cyclic voltametric behavior closely matches that of commercial gold electrodes, demonstrating the favorable electrochemical activity and reversibility of the elastic gold electrodes.

The cyclic voltammetry (CV) curve displays the characteristic redox profile of gold, with highly pronounced features. The sharp reduction peak and distinct oxidation peak confirm that the elastic gold electrode exhibits the same excellent electrochemical performance as commercially available rigid gold electrodes. Its strong current response demonstrates that the conductive network formed by gold nanowires (AuNWs) offers outstanding conductivity, facilitating efficient electron collection and transport. This result highlights that the elastic gold electrode is an excellent substrate for electrochemical sensing applications.

**Figure 5:**
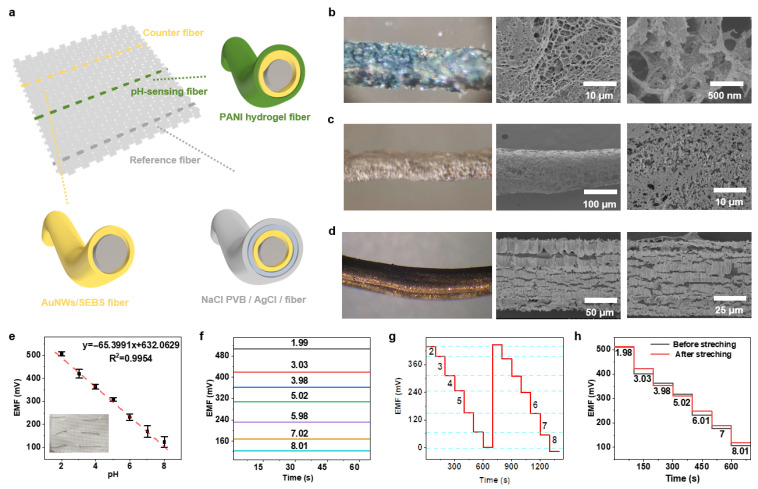
Performance evaluation of the PANI-PA-PVA hydrogel-based wearable pH sensor. (**a**) Schematic illustration of the three-electrode hydrogel sensor system. (**b**–**d**) SEM images of the working, reference, and counter electrodes, showing uniform coating and stable surface morphology. (**e**,**f**) pH response curves and calibration plots, demonstrating linear super-Nernstian sensitivity across the physiological sweat pH range. (**g**) Cyclic stability tests from pH 2–8, confirming excellent reproducibility and long-term reliability. (**h**) Repeatability analysis before and after mechanical stretching, indicating robust pH sensing performance under deformation.

Figure 5a illustrates the overall layout of the sensing unit, where three functional fibers are integrated in parallel to form a fabric sensor. The schematic clearly identifies each component: the elastic gold electrode fiber, which performs the counter electrode function; the Ag/AgCl reference electrode fiber, providing a stable potential reference; and the core working electrode fiber, encapsulated by the PANI-PA-PVA hydrogel. This design effectively separates and integrates the sensing, conductive, and reference functions across distinct fiber units, ensuring stable and reliable signal acquisition. The enlarged cross-sectional view on the right provides a detailed look at the internal composite structure of each fiber, offering a clear model of its operational mechanism.

To accommodate the deformation of human skin, the reference electrode must maintain structural or morphological integrity after undergoing mechanical strain. As shown in Figure S12, the surface morphology of the reference electrode exhibited minimal alteration following 125% stretching, demonstrating the stretchability of our fabricated elastic reference electrode. Crucially, for reference electrodes, maintaining potential accuracy under all conditions remains paramount. During testing, pH variations did not affect the reference electrode’s accuracy (Figure S13). We tested the fabricated elastic reference electrode using PBS buffer solutions of varying pH levels. Results confirmed excellent stability: across pH changes from 2 to 8, the potential variation was merely 5 mV—within acceptable limits. This demonstrates the fabricated elastic reference electrode possesses commendable stability and acid-alkali resistance. Similarly, to ensure sweat constituents do not interfere with the reference electrode, tests were conducted by adding various interfering substances to the PBS buffer solution (Figure S14). The final experiments demonstrated that common substances in sweat exert minimal influence on the reference electrode. This confirms our fabricated reference electrode is suitable for sweat monitoring.

To validate the feasibility of this design, we examined the microstructure of the materials. Figure 5b–d show optical microscope and SEM images of the three fiber electrodes. Figure 5b presents the PANI-PA-PVA hydrogel electrode fiber, revealing an interconnected porous network structure at multiple SEM magnifications. This high specific surface area facilitates rapid proton transfer and reaction, enabling sensitive and fast pH sensing. Figure 5c shows the Ag/AgCl/PVB reference electrode fiber, where the microstructure displays a stable surface formed by densely packed granular Ag/AgCl particles. During testing, sulphides or proteins within sweat composition may corrode the Ag/AgCl surface, causing drift in the reference potential. The PVB coating acts as a physical barrier, effectively isolating these interfering substances and significantly enhancing the long-term stability of the reference electrode. Furthermore, PVB permits the passage of small ions present in sweat while blocking macromolecules and colloidal substances. The incorporation of NaCl within the PVB coating ensures that ionic strength within the PVB membrane is predominantly governed by the fixed NaCl concentration. This stabilises the liquid junction potential between the PVB membrane and external sweat, shielding the entire reference system potential from fluctuations in external sweat Cl^-^ concentration. Consequently, stability and reliability are markedly enhanced in practical detection environments. Figure 5d illustrates the morphology of the counter electrode fiber, which consists of a wrinkled gold film, enhancing the surface area and providing a stable conductive interface.

To ensure accurate body fluid measurements, preliminary tests were conducted using PBS buffer solution before applying the flexible wearable sweat sensor for real-time human sweat monitoring. A standard curve was established, yielding a slope of 65.3991 mV pH^−1^ and a correlation coefficient of 0.9954, which conforms to the Nernst equation (Figure 5e,f).

The cyclic stability of the PANI-PA-PVA fiber electrode was evaluated through two consecutive measurements within a pH range of 2.00–8.00 (Figure 5g). The results demonstrate the PANI-PA-PVA electrode exhibits outstanding potential reproducibility, indicating its ability to maintain stable performance during cyclic testing. This confirms the suitability of the PANI-PA-PVA fiber electrode for continuous monitoring, enabling its application in subsequent practical, body-worn continuous sweat testing.

The mechanical deformation caused by the motion of human joints necessitates that skin-contact sensors preserve their electrochemical integrity despite being subjected to stretching and strain. To assess the mechanical durability of the PANI-PA-PVA sensor, the sensor was subjected to a degree of stretching in PBS buffer solution, with electrochemical tests conducted both before and after stretching (Figure 5h). Electrochemical testing demonstrated the sensor’s high sensitivity (63 mV pH^−1^) and excellent stability under mechanical stress, with the Nernstian slope showing minimal change from 63 mV pH^−1^ (R^2^ = 0.9984) before bending to 62 mV pH^−1^ (R^2^ = 0.9914) afterward. This confirms the PANI-PA-PVA material’s superior tensile durability.

**Figure 6:**
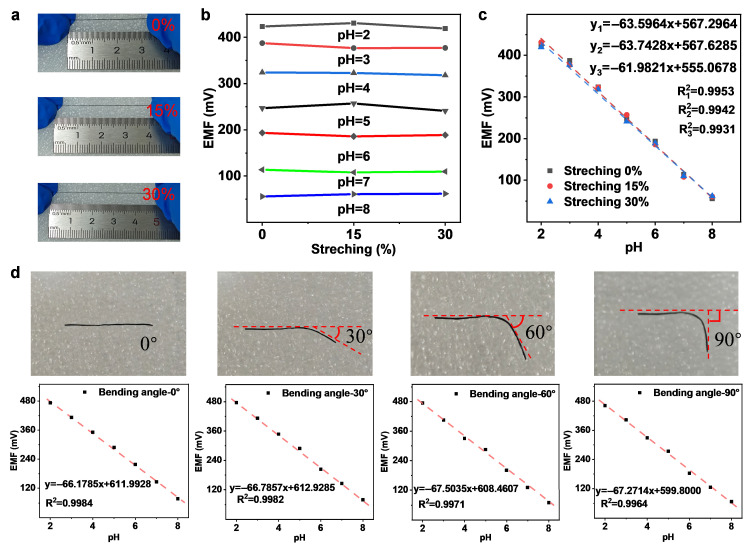
Mechanical durability of stretchable hydrogel-based electrodes. (**a**–**c**) Potentiometric pH responses after applying tensile strains of 0%, 15%, and 30%, demonstrating consistent sensitivity and minimal signal decay under increasing elongation. (**d**) Potentiometric pH responses under bending at 0°, 30°, 60°, and 90°, confirming stable electrochemical performance and excellent flexibility across different deformation states.

Additionally, we tested the sensitivity of the prepared PANI-PA-PVA sensor under stretching conditions. At a stretching ratio of 115%, the sensor still exhibited a good Nernstian response, with a slope of 63.7428 mV pH^−1^ and a correlation coefficient of 0.9942. At a stretching ratio of 130%, the slope was 61.9821 mV pH^−1^ with a correlation coefficient of 0.9931. In the unstretched state, the sensor exhibited a slope of 63.5664 mV pH^−1^ and a correlation coefficient of 0.9954 (Figure 6a–c). These results demonstrate that the PANI-PA-PVA hydrogel retains good conductivity under stretching conditions, and the wrinkled gold layer does not fracture during stretching. To validate this, we directly stretched the fiber-based pH working electrode to 100% strain. Under this condition, only minor cracks were observed on the fiber surface, but the PANI-PA-PVA membrane maintained its structural integrity, and the wrinkled gold layer stretched without breaking (Figure S15). Upon releasing the strain, the minimal structural changes in the PANI-PA-PVA membrane were restored. This explains the minor sensitivity changes observed between the stretched and initial states.

A pivotal assessment of the wearable sensor’s operational robustness involved characterizing its electrochemical sensitivity when subjected to mechanical deformation analogous to joint flexion. The investigation, summarized in Figure 6d, quantified the Nernstian response across a spectrum of folding angles (0°, 30°, 60°, 90°) over a pH range of 2–8. Analysis of the acquired data indicates that the sensor’s sensitivity, represented by the slope, displays exceptional invariance. The recorded values of 66.18 mV pH^−1^ (0°), 66.78 mV pH^−1^ (30°), 67.50 mV pH^−1^ (60°), and 67.27 mV pH^−1^ (90°) demonstrate that the maximum observed deviation is minuscule. Concurrently, the high correlation coefficients (R^2^), which remain above 0.9964 even at the most severe 90° bend, confirm that the linearity of the electrochemical response is uncompromised. The collective dataset allows us to conclude that there is no statistically significant correlation between the folding angle and the sensor’s sensitivity. The observed stability suggests that the constitutive materials effectively mitigate stress-induced alterations in electrochemical activity. Consequently, the sensor’s precision and reproducibility for continuous sweat pH monitoring, for instance during elbow flexion, are convincingly validated. To validate the mechanical stability of the PANI-PA-PVA hydrogel sensor in wearable applications, we systematically evaluated its electrochemical performance after different bending fatigue cycles (0, 30, 50, 100 cycles) (as shown in the Figure S16). The sensor exhibited excellent potential response under all conditions. Its pH sensitivity remained highly stable even after 100 bending cycles (0 cycles: −66.83 mV pH^−1^; 100 cycles: −68.21 mV pH^−1^), and the linearity coefficient (R^2^) of all calibration curves exceeded 0.99. These results conclusively demonstrate that the hydrogel sensor can withstand repeated mechanical deformation while maintaining stable core sensing functionality, meeting the requirements for wearable devices.

In summary, the PANI-PA-PVA-based sensor demonstrates a compelling profile for practical wearable applications, characterized by its exceptional bending resistance, superior tensile durability, and reliable stability under diverse operating conditions. This combination of mechanical robustness and electrochemical reliability is crucial for achieving accurate, real-time monitoring of dynamic physiological biomarkers such as sweat pH during human movement. Consequently, the sensor presents a highly promising platform for the development of next-generation wearable devices in the fields of personalized health monitoring and beyond.

### 2.5. Application of PANI-PA-PVA Hydrogel-Based Intelligent Wearable Sweat Sensors

**Figure 7:**
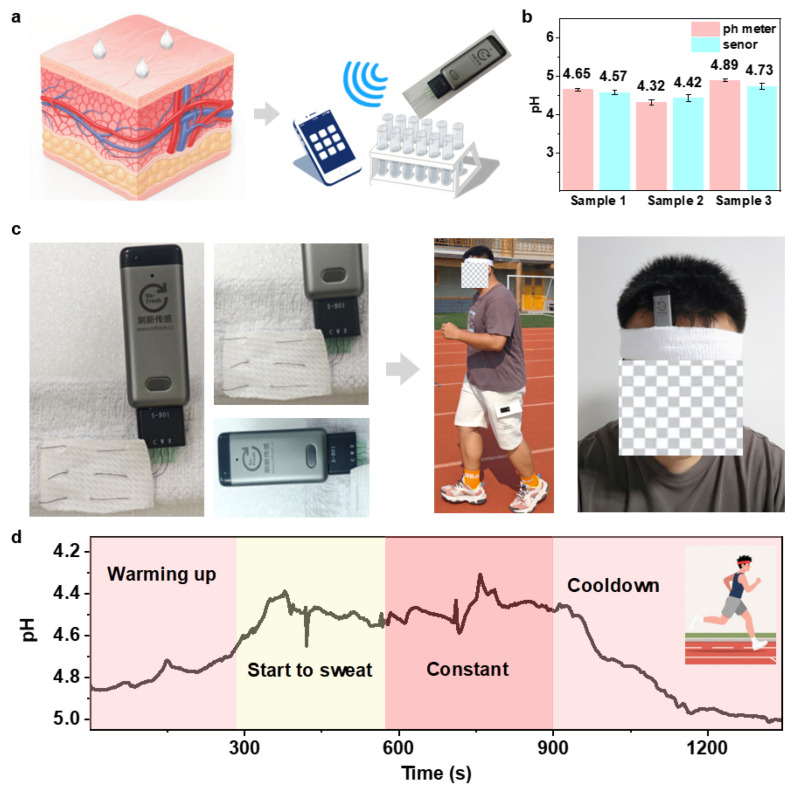
Smart wearable PANI-PA-PVA hydrogel sensor for non-invasive real-time sweat pH monitoring. (**a**) Schematic representation of human skin structure and movement, illustrating the dynamic environment for wearable sensing. (**b**) Comparison of sweat pH values measured by the hydrogel-based sensor and a commercial pH meter, showing excellent consistency and accuracy. (**c**) Structural configuration of the wearable device, consisting of a three-electrode hydrogel sensor integrated with a portable electrochemical workstation, Bluetooth module, and mobile interface; photographs of volunteers wearing the device during exercise demonstrate practical applicability. (**d**) Real-time monitoring of sweat pH during physical activity, confirming stable, responsive, and non-invasive sensing performance under dynamic conditions.

The wearable pH sensor is composed of a refreshable sensing micropotentiometer and a flexible, biocompatible PANI-PA-PVA pH chip. This assembly is designed for direct attachment to the skin, as depicted in Figure 7a, to facilitate the real-time monitoring of pH changes in sweat. Prior to each application, the sensor requires calibration prior to use. To rigorously evaluate the accuracy of its real-time measurements, a comparative analysis was conducted using three different sweat samples, where the data acquired by the PANI-PA-PVA sensor were benchmarked against readings from a standard pH meter. The results indicated a high degree of measurement precision, with only minimal discrepancies observed in the pH readings (Figure 7b). The stability of the sensor’s performance was further corroborated by a post-test calibration. In a practical demonstration of its functionality, the fully integrated sensor system was affixed to a subject’s forehead using an elastic bandage during a running exercise. This setup enabled continuous sweat analysis, with the collected data being wirelessly transmitted to a smartphone and displayed in real-time on a custom-developed mobile application (Figure 7c).

The real-time monitoring data of sweat pH, corresponding to the subject’s physiological response during exercise, is presented in Figure 7d. Initially, the potentiometric signal showed no significant fluctuations. After approximately 5 min of running at a speed of 10 km h^−1^, a young male subject began to sweat profusely. This physiological event was immediately detected by the wearable PANI-PA-PVA pH sensor as a pronounced decrease in the measured potential. As the subject continued running at a steady state, the sensor became fully saturated with sweat, leading to the stabilization of the electrochemical potential (EMF) signal, which then remained constant. This observed trend is consistent with established principles of exercise physiology: intense physical activity can induce a transient state of anaerobic respiration in skeletal muscles due to a relative oxygen deficit, resulting in the production and subsequent secretion of lactic acid into sweat, thereby lowering its pH. The accumulation of lactic acid lowers blood pH, which is closely linked to changes in sweat pH [36,37,38]. During exercise, the EMF signals detected in young males ranged from 248 to 200 mV, corresponding to a pH range of 4.2 to 5.0, which falls within the normal sweat pH range of 4.0–8.0. These results indicate that the sensor can accurately monitor pH changes in real-time during exercise.

The PANI-PA-PVA hydrogel-based wearable sensor has been successfully applied for real-time sweat monitoring, with practical value in fitness and health monitoring. However, there are areas for improvement: factors such as unstable sweat flow during exercise, evaporation interference, and mixing of new and old sweat can affect accuracy. Additionally, fluctuations in skin temperature may cause measurement errors. Future enhancements will include integrating an array of sensors with a microfluidic chip to automatically collect sweat through microchannels, separate new and old samples, and detect multiple indicators (such as ions and small molecule metabolites) to reduce errors. Adding a miniature temperature sensor and dynamic compensation algorithm for real-time monitoring of skin contact surface temperature changes and automatic data calibration will further improve measurement precision and stability, providing more reliable data for personalized medicine.

## 3. Conclusions

In summary, we developed a flexible, fiber-integrated pH sensor based on a PANI-PA-PVA hydrogel, demonstrating that rational hydrogel design can simultaneously achieve mechanical resilience and superior electrochemical performance. The cross-linked and porous interpenetrating polymer network facilitated efficient ion and electron transport, yielding a super-Nernstian sensitivity of 68.8 mV·pH^−1^, ultrafast response (<10 s) within the sweat-relevant range, and excellent long-term stability with negligible potential drift. The sensor maintained consistent pH readings under repeated bending and stretching, confirming its robustness for wearable operation. Integration with elastic gold fibers and Ag/AgCl reference electrodes enabled accurate on-skin sweat-pH monitoring, with results comparable to commercial pH meters during physical activity. Beyond quantitative performance, this work establishes a generalizable strategy for conductive-hydrogel design, where structural flexibility is synergistically coupled with electrochemical functionality. The PANI-PA-PVA hydrogel-based system therefore represents a promising platform for continuous, non-invasive physiological monitoring.

Nevertheless, some limitations remain. The current design primarily targets pH detection in controlled environments, and extended performance validation under variable sweat rates, temperatures, multiple subjects, and long-term skin contact is still needed. Future studies should focus on improving biocompatibility, miniaturization, and integration with wireless data modules, as well as extending this material platform to multi-analyte biosensing (e.g., ions, metabolites, and reactive species). These developments will accelerate the translation of PANI-based hydrogels into next-generation wearable health-monitoring systems for personalized medicine and early disease diagnostics.

## 4. Materials and Methods

### 4.1. Materials

Gold chloride trihydrate (HAuCl_4_·3H_2_O) and MBA were purchased from Adamas. Oleylamine (OA) was sourced from Sigma-Aldrich, while triisopropylsilane (TIPS), silicon oil, aniline (ANI), phytic acid (PA, 70 wt% aqueous solution), tetrahydrofuran (THF), ammonium chloride (NH_4_Cl), sodium chloride (NaCl), potassium chloride (KCl), calcium chloride (CaCl_2_), glucose, lactate, and ammonium persulfate (APS, ≥98%) polyvinyl butyral (PVB, 70,000–100,000 MW) were obtained from Aladdin. L-ascorbic acid (L-AA) and uric acid were purchased from Macklin. SEBS (G1651H) was obtained from Kraton. Hydrochloric acid (HCl), sulfuric acid (H_2_SO_4_), silver nitrate (AgNO_3_), sodium phosphate (Na_2_HPO_4_), potassium phosphate (KH_2_PO_4_), and anhydrous ethanol (EtOH) were sourced from Sinopharm Chemical Reagent Co., Ltd. (Beijing, China). Polyvinyl alcohol (PVA, 1750) was also supplied by Sinopharm Chemical Reagent Co., Ltd. After grinding, the gold-plated electrodes were treated with ultrasonic cleaning in deionized water for 30 s, followed by sequential ultrasonic cleaning in ethanol and deionized water to remove surface contaminants. All chemical reagents used in the experiment were of analytical grade purity, and deionized water was employed throughout the entire experimental process (resistivity > 18 MΩ·cm).

### 4.2. Material Characterization

The surface morphology and elemental composition of PANI-PA-PVA hydrogels were examined using a scanning electron microscope (SEM, SU8010, Hitachi High-Technologies Corporation, Tokyo, Japan) equipped with an energy-dispersive X-ray spectrometer (EDS). Fourier transform infrared spectroscopy (FTIR) was performed using an FTIR-650 spectrometer (Tianjin Guangdong Sci-Tech Development Co., Ltd., Tianjin, China) within the spectral range of 4000–400 cm^−1^. Gold nanowires were separated via a DH-1600 centrifuge (Shanghai Deyang Yibang Instrument Co., Ltd., Shanghai, China). Electrochemical performance testing was conducted at room temperature using a CHI660E electrochemical workstation (China CH Instruments Co., Ltd., Shanghai, China), employing a three-electrode system comprising a 3.0 mm diameter gold working electrode, a platinum wire counter electrode, and an Ag/AgCl reference electrode. pH measurements were performed using a commercial pH meter (PHS-3C, China Laishi, Shanghai, China). Human sweat testing employed a portable smart sensor manufactured by Shenzhen Refresh Biosensor Technology Co., Ltd. (Shenzhen, China) Data acquisition was performed via a portable workstation equipped with a wireless USB platform. Gold nanowires were observed using a transmission electron microscope (TEM, JEOL JEM-2100 plus, Tokyo, Japan).

### 4.3. Preparation of PANI-PA-PVA Hydrogel

The preparation of the PANI-PA-PVA hydrogels followed the procedure adapted from previous studies [39]. First, two solutions (A and B) were prepared. Solution A was made by dissolving 7 wt% polyvinyl alcohol (PVA) in water at 90 °C. After cooling to room temperature, 3.55 mL of the PVA solution was transferred, and 905 µL of aniline (ANI) and 715 µL of phytic acid (PA) were added and dissolved to form Solution A. Solution B was prepared by dissolving 570 mg of ammonium persulfate (APS) (APS/ANI = 0.12 mol%) in deionized water. After cooling both Solution A and Solution B to 0 °C, they were mixed thoroughly and left at room temperature for 12 h. The mixture then underwent three freeze–thaw cycles (freezing at −20 °C for 12 h and thawing at room temperature for 12 h). The prepared hydrogel was immersed in deionised water for 12 h to remove excess phytate, yielding the final PANI-PA-PVA hydrogel.

### 4.4. Electrochemical Characterization

Electrochemical characterisation was performed at room temperature using a CHI660 electrochemical workstation and a three-electrode system. The working electrode consisted of three independently prepared components: a PANI-PA-PVA-modified gold electrode, a platinum counter electrode, and an Ag/AgCl reference electrode. pH testing was conducted in PBS buffer solutions over a range of 2.00 to 8.00, and the change in electric potential (EMF) between the working and reference electrodes was recorded. Open circuit potential (OCP) measurements were taken in PBS buffer solutions across the pH range of 2.00 to 8.00. The electrodes were immersed in the solution to assess their response to changes in H+ concentration, and the stabilizing potential was recorded. Cyclic voltammetry (CV) was conducted in 1 M H_2_SO_4_ with potential sweeps ranging from −0.2 V to 0.8 V at scan rates of 2.5 to 150 mV s^−1^ to confirm the electrochemical behavior of the PANI-PA-PVA electrodes. Electrochemical impedance spectroscopy (EIS) was performed using 5 mM K_3_[Fe(CN)_6_] to evaluate the impedance characteristics of the electrodes over a frequency range of 1 MHz to 0.01 Hz, with an amplitude of 10 mV. Fitting of electrochemical impedance spectra was performed using Zview software (Version 2.70, Scribner Associates Inc., North Carolina, USA). The sensor’s response to potential interference from light, gases, temperature fluctuations, ions (Na^+^, K^+^, NH_4_^+^, Ca^2+^), and biomolecules (glucose, lactate, uric acid, and L-ascorbic acid) was also assessed. For sensitivity testing, PANI-PA-PVA electrodes were immersed in PBS buffer solutions (pH 2.00–8.00), and the potential responses to pH changes were recorded after the OCP signal stabilized.

### 4.5. Preparation of Stretchable Au Fiber Electrode

The preparation of dry-spun stretchable gold electrodes was adapted from published literature [40]. First, 3.4 mL of oleylamine, 100 mg of chloroauric acid trihydrate, and 4.7 mL of triisopropylsilane were sequentially added to 40 mL of hexane and gently shaken until the solution turned orange and was well mixed. After standing at room temperature for 48 h, the solution changed to a dark red color. Three times the volume of ethanol was added to the stock solution, and a black precipitate formed upon centrifugation at 4000 rpm for 5 min. The precipitate was then dissolved in 4 mL of tetrahydrofuran by shaking. SEBS and silicone oil were sequentially added, with the final solution having a composition of SEBS/silicone oil/AuNWs/tetrahydrofuran (10/1/1/0.1 by weight/weight/weight/volume). The mixture was vigorously shaken for 2 d, then transferred into a 1 mL syringe with a 25-gauge needle. The solution was extruded via a syringe pump at a fixed rate of 1 mL h^−1^ and rapidly dried in air to form AuNWs/SEBS fibers. The dried fibers were pre-stretched to 300% and immersed in a solution containing 2.4 mL of ethanol, 0.1 mL of 60 mM 4-mercaptobenzoic acid, 440 µL of 150 mM chloroauric acid trihydrate, 2.2 mL of H_2_O, and 0.4 mL of 400 mM L-ascorbic acid for 8 min. After immersion, the fibers were repeatedly washed with deionized water and then immersed in a 25 µM sodium borohydride solution in ice-water for 10 min to remove excess 4-mercaptobenzoic acid. The prepared fibers were washed again with deionized water, dried at room temperature for at least 2 h, and slowly released to their original length to form the dry-spun stretchable gold electrodes.

### 4.6. Preparation of the Fiber Sensor

#### 4.6.1. Preparation of Ag/AgCl Reference Fiber Electrode

The Ag/AgCl reference electrode was prepared using dry-spun gold fibers. First, a layer of silver was electroplated onto the gold fibers by applying a voltage of −0.3 V for 60 s in a 5 mM AgNO_3_/1 M KNO_3_ solution using an amperometric i-t curve. The silver layer was then chlorinated using cyclic voltammetry in a 10 mM KCl/0.1 M HCl solution, with four cycles from −0.15 V to 1.05 V at a scan rate of 0.05 V s^−1^. After allowing the electrode to dry, a portion of the reference electrode was immersed in a mixed solution prepared by dissolving 78 mg of polyvinyl butyral (PVB) and 50 mg of NaCl in 1 mL of methanol.

#### 4.6.2. Preparation of PANI-PA-PVA Hydrogel Fiber Electrode

The PANI-PA-PVA hydrogel solution was coated onto the surface of the gold fibers using the drop-casting method to form a dense membrane. The sample was left at room temperature for 12 h, followed by three freeze–thaw cycles (freezing at −20 °C for 12 h and thawing at room temperature for 12 h). The hydrogel-coated gold fiber electrode was then immersed in pH = 7, 20 mM PBS buffer solution for 12 h, removed, and rinsed briefly with deionized water before use.

#### 4.6.3. Preparation of Wearable Sweat Fiber Sensor

Due to the small size of the gold fiber electrodes (1 mm in diameter), they were woven into T-shirt fabric to create a wearable sweat pH sensor. Three electrodes were sewn into the fabric, maintaining equal spacing between them. The electrodes were connected to the electrochemical workstation using commercial conductive wires. After each test, the working electrode was placed in a pH = 7, 20 mM PBS buffer solution, while the reference and counter electrodes were left to dry at room temperature for the next test.

### 4.7. Characterization of the Sensing Fibers

The performance of the pH sensors based on Au fibers was investigated by monitoring changes in open circuit potential (OCP) in real time under different stretching states (0% to 30%), bending angles (0° to 90°), and bending cycles in PBS buffer solutions with varying pH values. All electrochemical tests were performed using a CHI660E electrochemical workstation. The fiber structure was characterized using scanning electron microscopy (SEM, SU 8010, Hitachi High-Technologies Corporation, Tokyo, Japan), and the morphology of oleylamine (OA)-capped gold nanowires (AuNWs) was analyzed using transmission electron microscopy (TEM, JEOL JEM-2100plus, Hitachi High-Technologies Corporation, Tokyo, Japan).

### 4.8. On-Body Measurements

The smart textile sweat pH sensor was integrated by weaving a fiber-based three-electrode sensor into the textile and securing it to the participant’s forehead using a headband. The participant’s skin was first cleaned with alcohol, and then the headband with the flexible sensor was worn. During the test, the volunteer (a healthy 25-year-old male) performed a 5 min warm-up, followed by 10 min of vigorous running, and concluded with 5 min of rest. OCP was recorded throughout to monitor sweat pH levels. All electrochemical measurements were conducted using the BIOSYS P20 electrochemical workstation (Refresh, Shenzhen Refresh Biosensing Technology Co., Ltd., Shenzhen, China), with data wirelessly transmitted via Bluetooth from the sensor to a custom mobile application.

## Data Availability

The original contributions presented in this study are included in the article/Supplementary Materials. Further inquiries can be directed to the corresponding author(s).

## Supplementary Materials

The following supporting information can be downloaded at: https://www.mdpi.com/article/10.3390/gels11110853/s1, Figure S1. (a–d) SEM images of PVA at magnifications of 50 µm, 25 µm, 5 µm, and 250nm; Figure S2. (a,b) pH sensing response of bare gold electrodes; Figure S3. (a,b) pH sensing response of PANI only electrodes; Figure S4. Response time of PANI-PA-PVA electrode to pH; Figure S5. (a,b) Average response time to pH for PANI-PA-PVA electrodes and PANI-only electrodes; Figure S6. (a,b) Interference Testing of PANI-PA-PVA Hydrogel Electrodes by Common Gases and Everyday Environmental Conditions; Figure S7. Protonic Acid Doping Mechanism in PANI-PA-PVA Hydrogels; Figure S8. (a,b) Cyclic voltammetry curves of PANI only electrode and corresponding diffusion characteristics in 1 M H2SO4; Figure S9. Transmission electron microscopy (TEM) image of gold nanowires (AuNWs); Figure S10. Elastic dry-spun gold electrode fabrication process; Figure S11. Cyclic voltametric curve of elastic gold electrode; Figure S12. Scanning electron microscope images of the Ag/AgCl reference electrode in its unstretched state and after stretching to 125%; Figure S13. Stability of the reference electrode with respect to pH; Figure S14. Interference Testing of ions and small molecules in sweat on Ag/AgCl reference electrodes; Figure S15. Scanning electron microscope images of the working electrode: untreated, stretched to 125%, and after spring-back following stretching; Figure S16. (a,b) The pH response measured by the potentiometric method after 0, 30, 50, and 100 cycles demonstrated consistent sensitivity with minimal signal attenuation as the number of flexures increased; (c–f) Potentiometric pH responses measured at 0, 30, 50, and 100 bends confirm its stable electrochemical performance across varying deformation states; Table S1. Elemental content analysis by EDS of PANI-PA-PVA; Table S2. Comparison of the performances of different pH sensors [41,42,43,44,45,46,47,48].
