# Supplementary material for: Development and Electrochemical Performance of a PANI-PA-PVA Hydrogel-Based Flexible pH Fiber Sensor for Real-Time Sweat Monitoring
Source: Gels. 2025 Oct 25;11(11):853. doi: 10.3390/gels11110853 (PMC12652390; doi:10.3390/gels11110853)
Supplement: Supplementary file 1 [file gels-11-00853-s001.zip › gels-3938682-supplementary.pdf]

Supporting Information

DSupplementary Figures

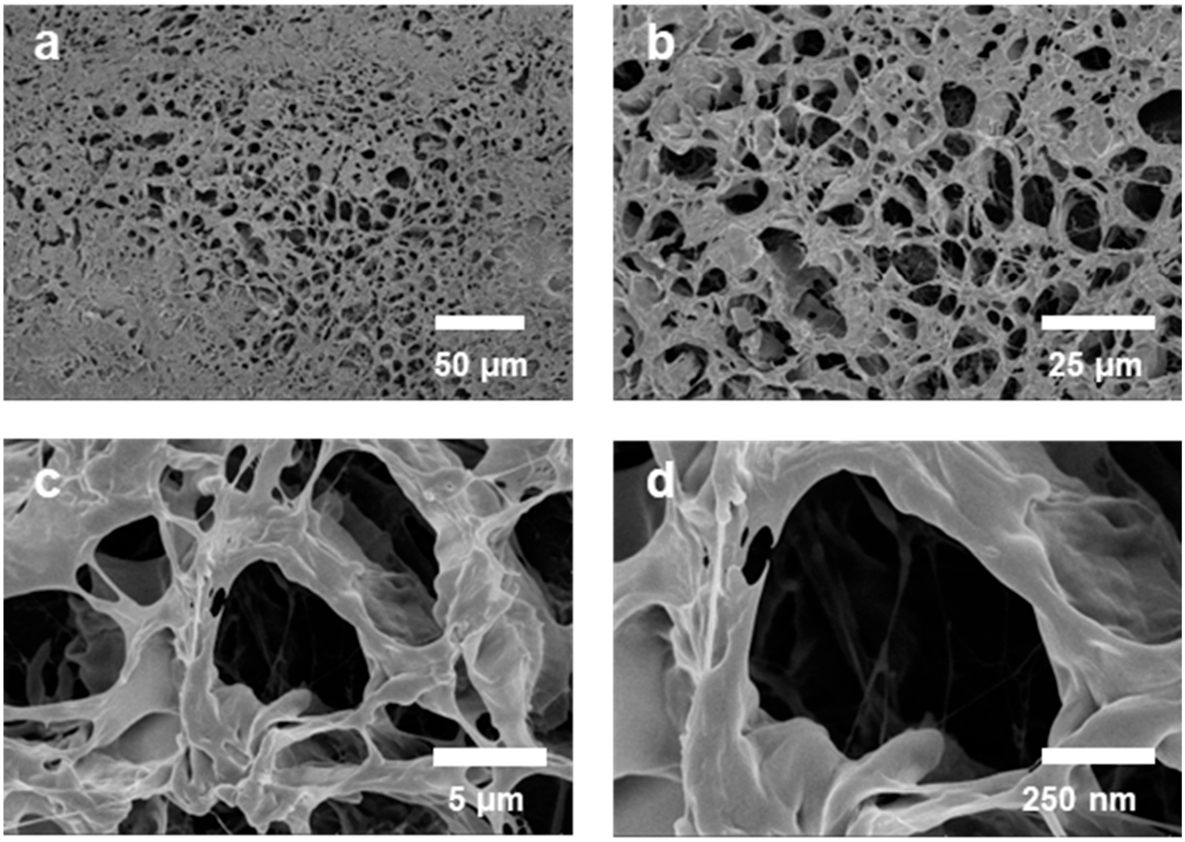

Figure S1. (a-d) SEM images of PVA at magnifications of 50 μm, 25 μm, 5 μm, and 250nm.

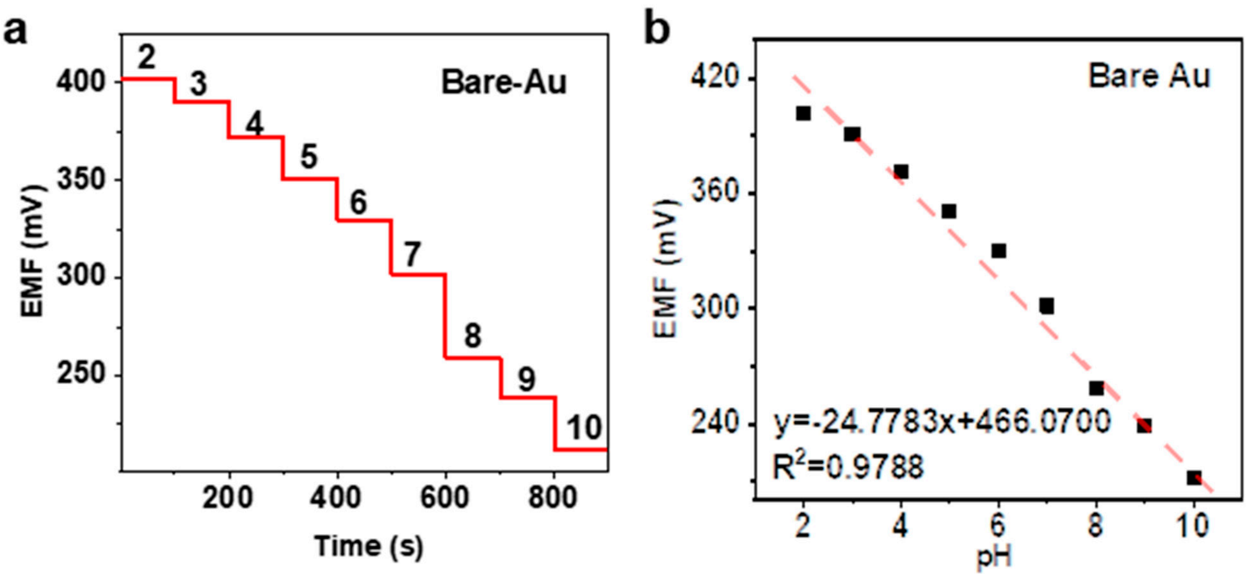

Figure S2. (a-b) pH sensing response of bare gold electrodes.

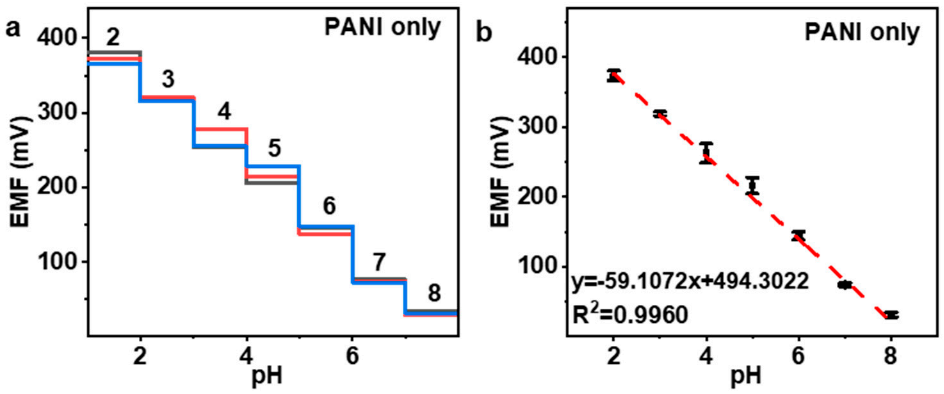

Figure S3. (a-b) pH sensing response of PANI only electrodes.

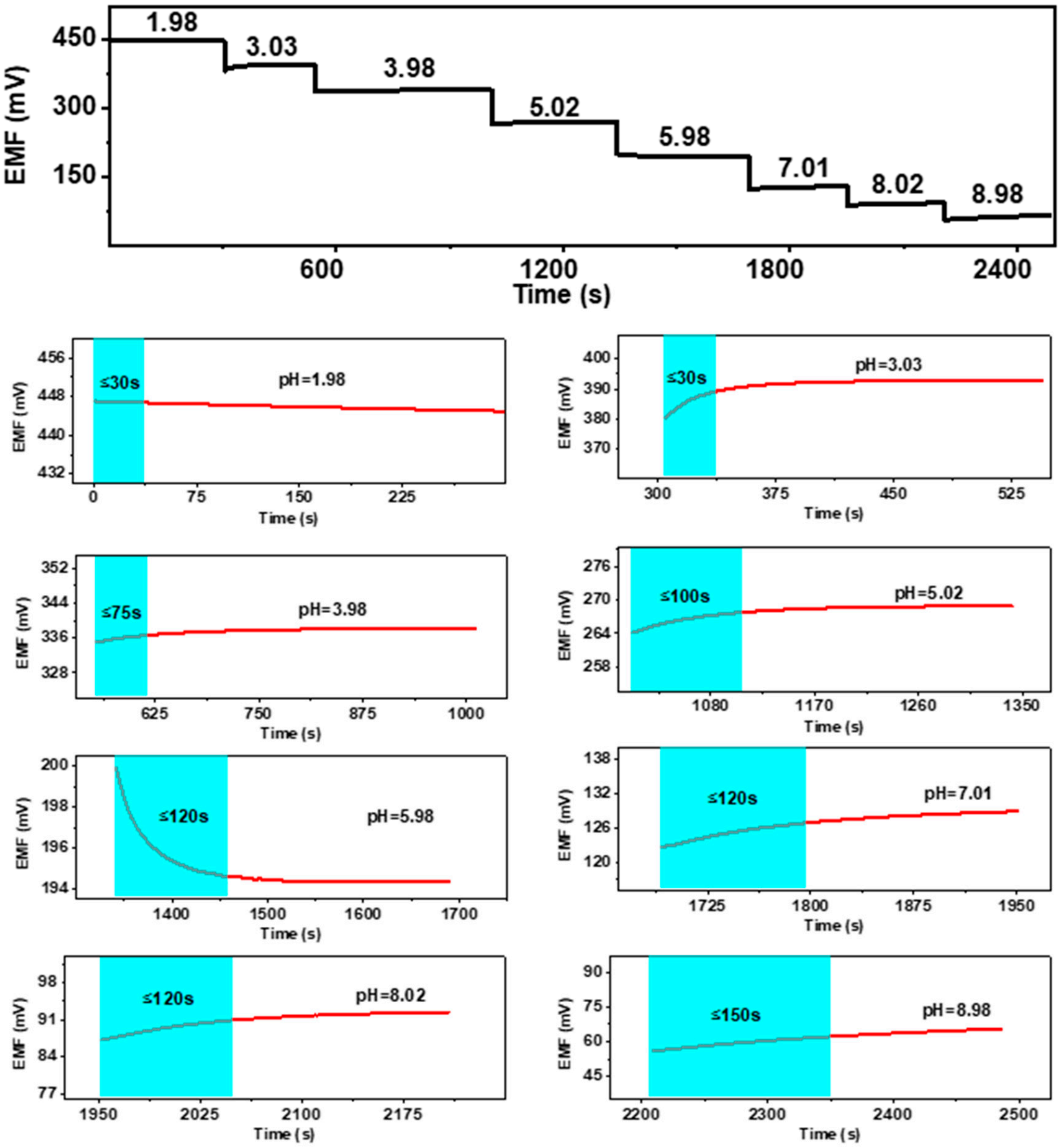

Figure S4. Response time of PANI-PA-PVA electrode to pH.

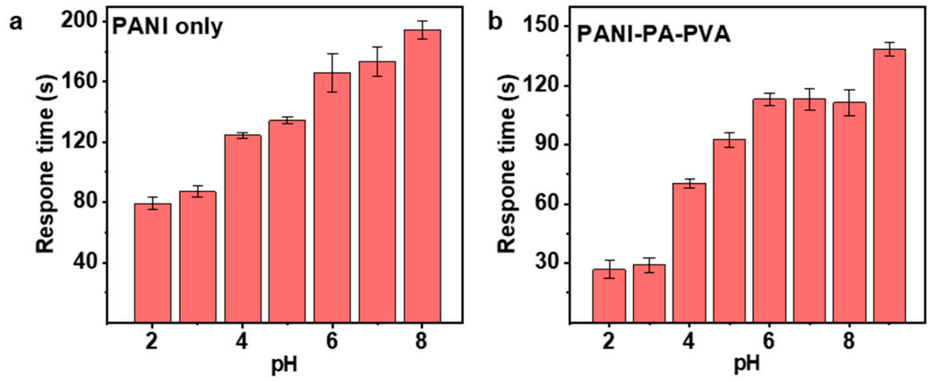

Figure S5. (a-b) Average response time to pH for PANI-PA-PVA electrodes and PANI-only electrodes.

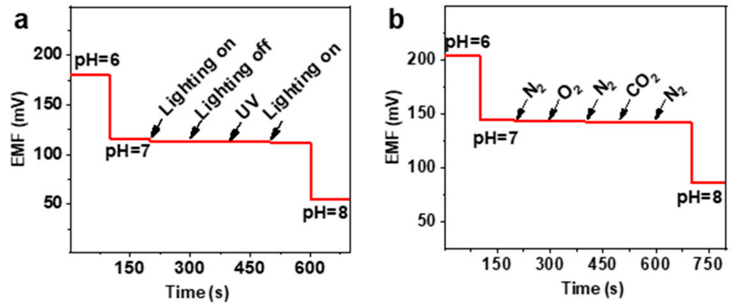

Figure S6. (a-b) Interference Testing of PANI-PA-PVA Hydrogel Electrodes by Common Gases and Everyday Environmental Conditions.

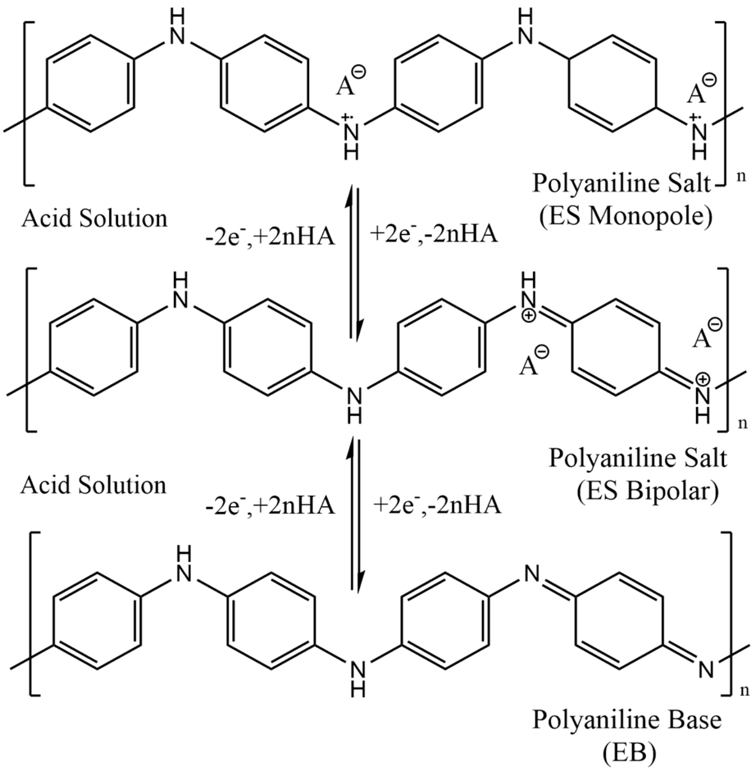

Figure S7. Protonic Acid Doping Mechanism in PANI-PA-PVA Hydrogels.

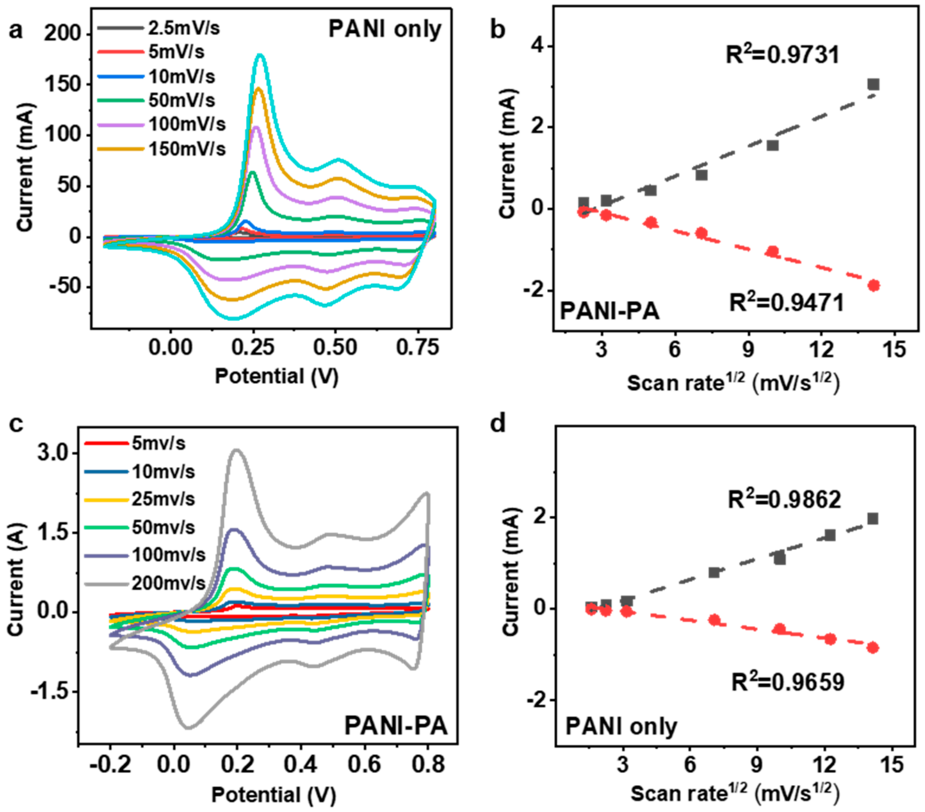

Figure S8. (a-b) Cyclic voltammetry curves of PANI only electrode and corresponding diffusion characteristics in 1 M H_2SO_4; (c-d) Cyclic voltammetry curves of PANI-PA electrode and

corresponding diffusion characteristics in 1 M H_2SO_4

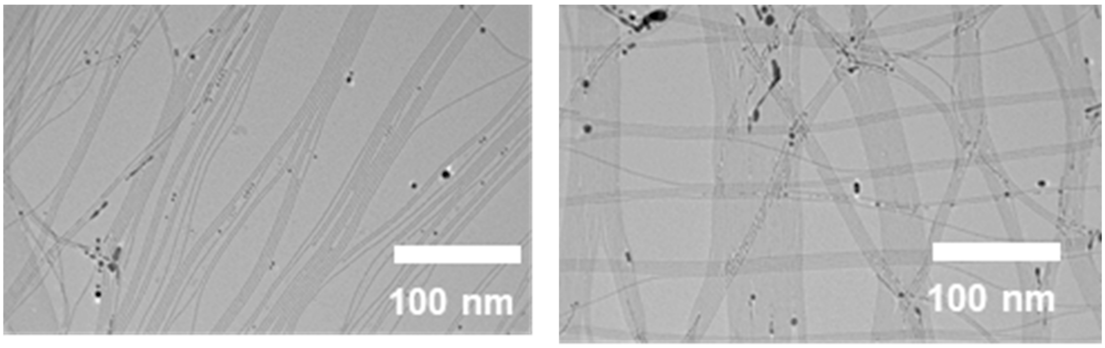

Figure S9. Transmission electron microscopy (TEM) image of gold nanowires (AuNWs).

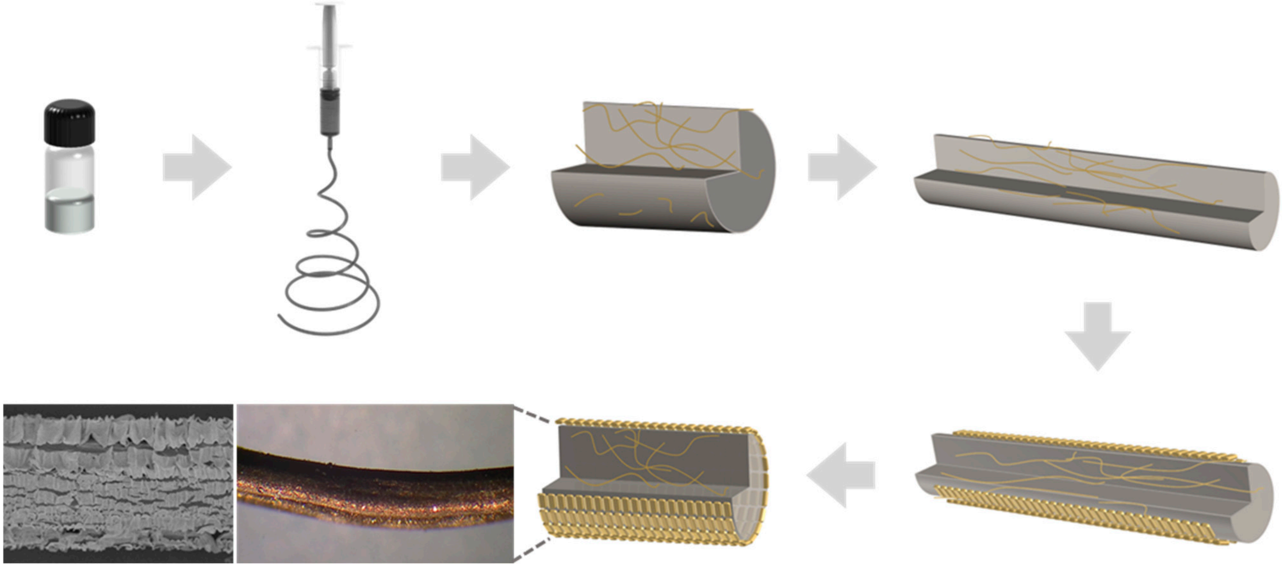

Figure S10. Elastic dry-spun gold electrode fabrication process.

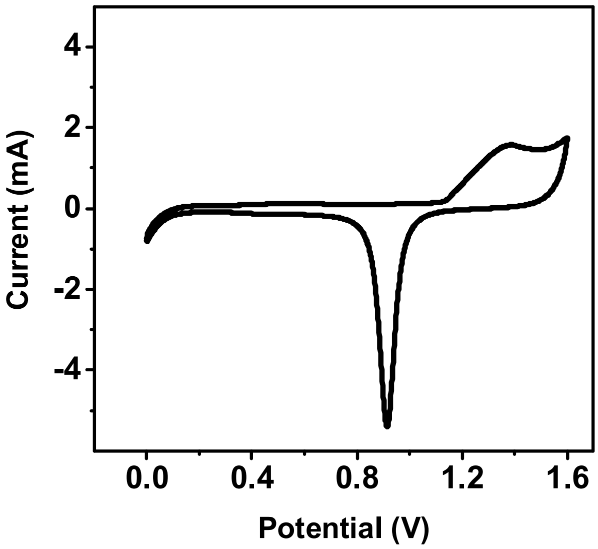

Figure S11. Cyclic voltammetric curve of elastic gold electrode.

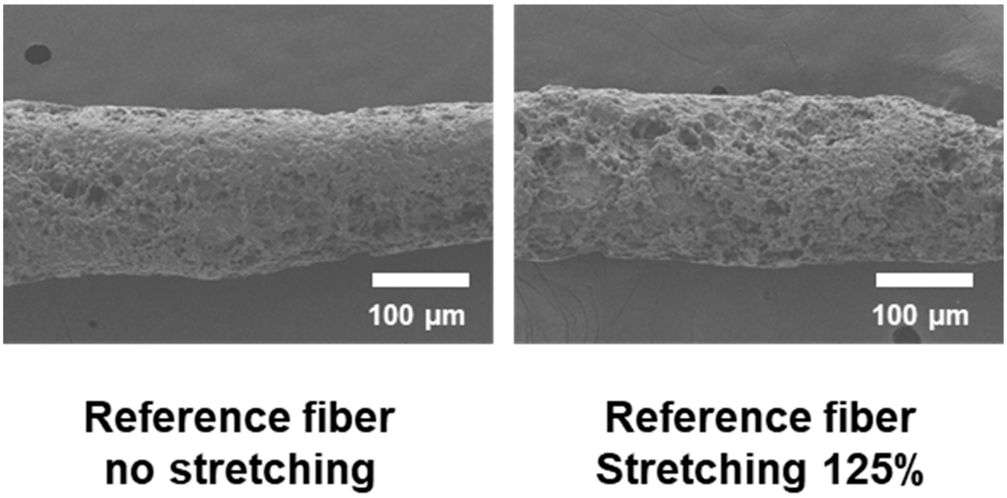

Figure S12. Scanning electron microscope images of the Ag/AgCl reference electrode in its unstretched state and after stretching to 125 %.

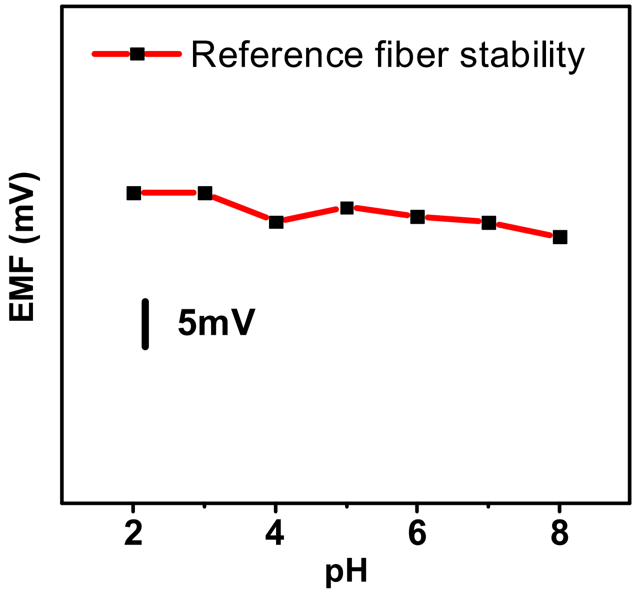

Figure S13. Stability of the reference electrode with respect to pH.

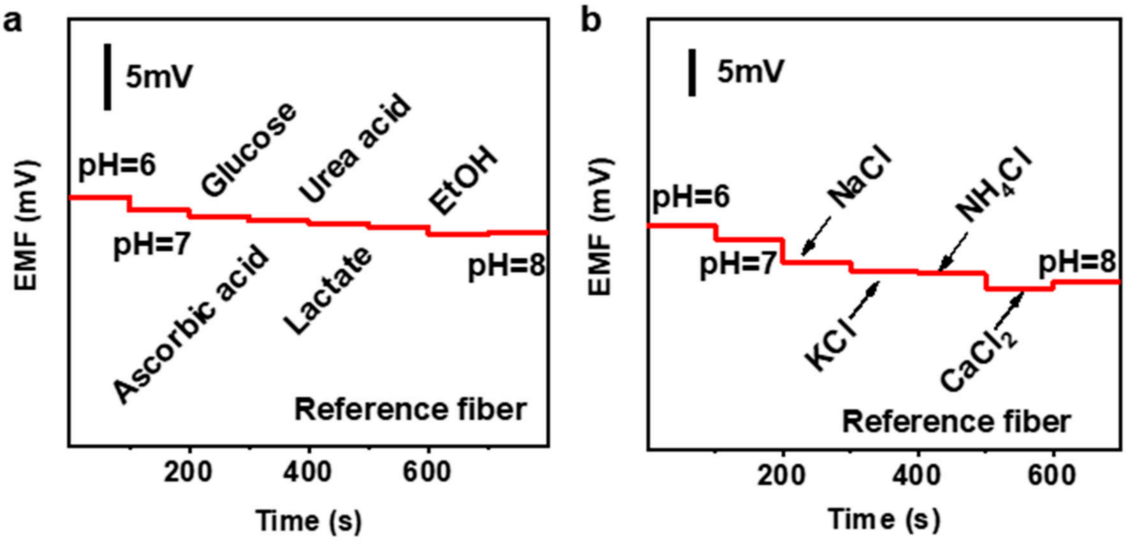

Figure S14. Interference Testing of ions and small molecules in sweat on Ag/AgCl reference electrodes.

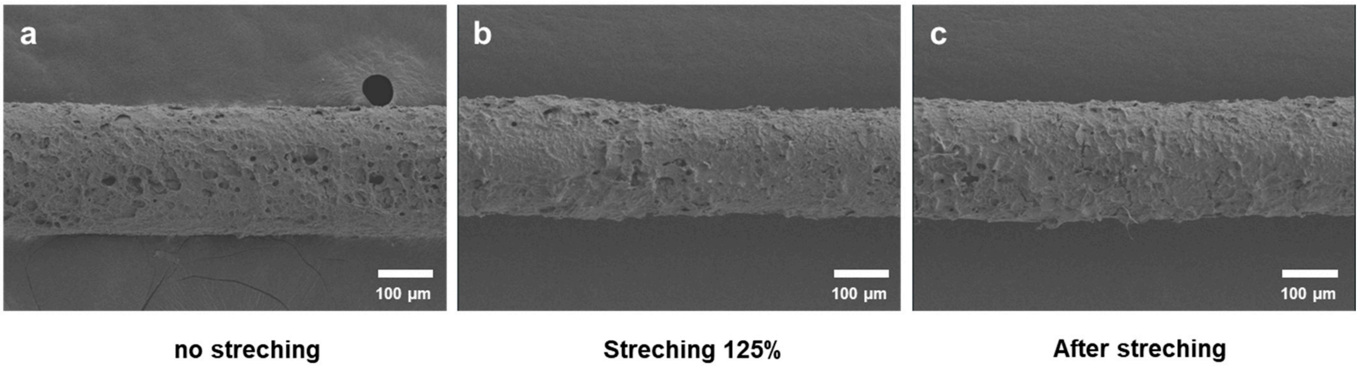

Figure S15. Scanning electron microscope images of the working electrode: untreated, stretched to 125 %, and after spring-back following stretching.

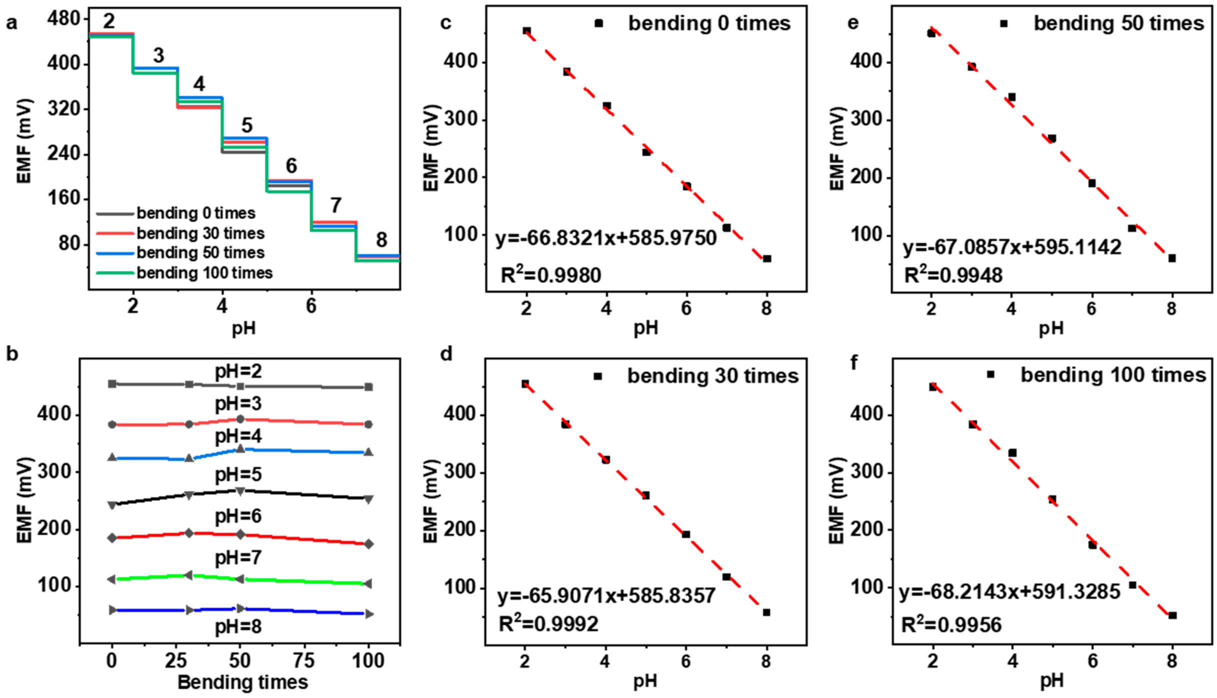

Figure S16. (a-b) The pH response measured by the potentiometric method after 0, 30, 50, and 100 cycles demonstrated consistent sensitivity with minimal signal attenuation as the number of flexures increased; (c-f) Potentiometric pH responses measured at 0, 30, 50, and 100 bends confirm its stable electrochemical performance across varying deformation states.

Supplementary Tables

Table S1 Elemental content analysis by EDS of PANI-PA-PVA.

Elt	Line	Intensity (c/s)	Cone	Units	Error 2-sig	MDL 3-sig	
C	Ka	826.17	55.410	wt.%	0.395	0.133	
N	Ka	3.69	1.538	wt.%	0.445	0.635	
O	Ka	299.08	33.504	wt.%	0.411	0.206	
P	Ka	703.33	9.508	wt.%	0.076	0.041	
			100.000	wt.%			Total

Table S2 Comparison of the performances of different pH sensors.

Active material	Sensitivity (mV/pH)	Response time (s)	Drift rate (mV/h)	pH range	Ref
PANI	59.2	-			[1]
PdO	60.6	15	1.6	4-10	[2]
IrO_2	47.54	-	-	4-8	[3]
3D PANI	69.33	7.75	0.1	4-9	[4]
PANI	62.3	250	-	2-8	[5]
PANI/PU	60	-	-	2-7	[6]

PANI film	63.72	-	-	1-12	[7]
PANI	-	-	-	2-12	[8]
PANI/G	53	15	-	3-10	[9]
PANI-PA-PVA	68.72	≤10	0.0925	2-9	This work

References

[1] Bandodkar A J, Hung V W S, Jia W, et al. Tattoo-based potentiometric ion-selective sensors for epidermal pH monitoring[J]. Analyst, 2013, 138(1): 123-128.

[2] Qin Y, Alam A U, Pan S, et al. Integrated water quality monitoring system with pH, free chlorine, and temperature sensors[J]. Sensors and Actuators B: Chemical, 2018, 255: 781-790.

[3] Zamora M L, Dominguez J M, Trujillo R M, et al. Potentiometric textile-based pH sensor[J]. Sensors and Actuators B: Chemical, 2018, 260: 601-608.

[4] Zhao Y, Yu Y, Zhao S, et al. Highly sensitive pH sensor based on flexible polyaniline matrix for synchronal sweat monitoring[J]. Microchemical Journal, 2023, 185: 108092.

[5] Park H J, Yoon J H, Lee K G, et al. Potentiometric performance of flexible pH sensor based on polyaniline nanofiber arrays[J]. Nano Convergence, 2019, 6(1): 9.

[6] Hou X, Zhou Y, Liu Y, et al. Coaxial electrospun flexible PANI/PU fibers as highly sensitive pH wearable sensor[J]. Journal of Materials Science, 2020, 55(33): 16033-16047.

[7] Zhu C, Xue H, Zhao H, et al. A dual-functional polyaniline film-based flexible electrochemical sensor for the detection of pH and lactate in sweat of the human body[J]. Talanta, 2022, 242: 123289.

[8] Wang E, Niu H, Li P, et al. Optical fiber F-P cavity pH sensor based on polyaniline reaction deposition film layer[J]. Measurement, 2025, 243: 116454.

[9] Mahinnezhad S, Emami H, Ketabi M, et al. Fully printed pH sensor based in carbon black/polyaniline nanocomposite[C]. 2021 IEEE Sensors, 2021: 1-4.
